# Advancements in Artificial Intelligence for the Diagnosis of Multidrug Resistance and Extensively Drug-Resistant Tuberculosis: A Comprehensive Review

**DOI:** 10.7759/cureus.60280

**Published:** 2024-05-14

**Authors:** Shanmuga Priya K, Anbumaran Parivakkam mani, Geethalakshmi S, Sankalp Yadav

**Affiliations:** 1 Department of Pulmonology, Faculty of Medicine, Sri Lalithambigai Medical College and Hospital, Dr MGR Educational and Research Institute, Chennai, IND; 2 Department of Respiratory Medicine, Saveetha Medical College and Hospital, Saveetha Institute of Medical and Technical Sciences, Saveetha University, Chennai, IND; 3 Department of Microbiology, Sri Lalithambigai Medical College and Hospital, Dr MGR Educational and Research Institute, Chennai, IND; 4 Department of Medicine, Shri Madan Lal Khurana Chest Clinic, New Delhi, IND

**Keywords:** mycobacterium tuberculosis (mtb), intestinal tb, multiple-drug resistant tuberculosis (mdr-tb), xdr-tb: extensively drug resistant tuberculosis, mdr tb, artificial intelligence

## Abstract

Tuberculosis (TB) remains a significant global health concern, particularly with the emergence of multidrug-resistant tuberculosis (MDR-TB) and extensively drug-resistant tuberculosis (XDR-TB). Traditional methods for diagnosing drug resistance in TB are time-consuming and often lack accuracy, leading to delays in appropriate treatment initiation and exacerbating the spread of drug-resistant strains. In recent years, artificial intelligence (AI) techniques have shown promise in revolutionizing TB diagnosis, offering rapid and accurate identification of drug-resistant strains. This comprehensive review explores the latest advancements in AI applications for the diagnosis of MDR-TB and XDR-TB. We discuss the various AI algorithms and methodologies employed, including machine learning, deep learning, and ensemble techniques, and their comparative performances in TB diagnosis. Furthermore, we examine the integration of AI with novel diagnostic modalities such as whole-genome sequencing, molecular assays, and radiological imaging, enhancing the accuracy and efficiency of TB diagnosis. Challenges and limitations surrounding the implementation of AI in TB diagnosis, such as data availability, algorithm interpretability, and regulatory considerations, are also addressed. Finally, we highlight future directions and opportunities for the integration of AI into routine clinical practice for combating drug-resistant TB, ultimately contributing to improved patient outcomes and enhanced global TB control efforts.

## Introduction and background

Tuberculosis (TB) is a common infectious disease caused by *Mycobacterium tuberculosis* [1,2]. In modern developing countries, TB is seen as a global threat to public health and the economy. According to a recent World Health Organization (WHO) 2023 report, around 1.3 million individuals succumbed to TB in 2022, including 1,67,000 HIV (human deficiency virus) cases [3-7]. Worldwide, scenarios report that TB is the second leading infectious killer after the pandemic, which is present in all countries and reported in all age groups. Also, the WHO report estimated that approximately 10.6 million people were diagnosed with confirmed cases of TB infection globally; among them, 5.8 million individuals were male, 3.5 million cases were female, and 1.3 million cases were children [3,8]. Although TB is a curable and preventable disease, antimicrobial resistance (AMR) is still a constant threat to global healthcare services, which has a significant toll on the economic burden of these nations [9,10].

In the current era, there has been a noticeable surge in the mortality rate attributed to multidrug-resistant tuberculosis (MDR-TB), resulting in a significant burden. In 2022, a statistical survey shows that, out of every five individuals diagnosed with TB, only two cases would have drug-resistant strains, presenting a significant challenge for TB management [11-14]. To the guidelines established by the WHO, TB must be confirmed bacteriologically to identify multidrug-resistant or rifampicin-resistant tuberculosis (MDR/RR-TB) [15,16]. It is concerning that drug-resistant tuberculosis (DR-TB), which includes extensively drug-resistant tuberculosis (XDR-TB) and MDR-TB, is becoming more prevalent [17]. To treat DR-TB prolonged, treatment time and the use of second-line anti-tuberculosis (ATTB) medications are indicated. On the other hand, abuse of these medications over time might damage several organs [17,18]. The efficacy of ATTB drugs must be accurately predicted and promptly monitored. For improved patient outcomes, a decreased risk of medication-related side effects and resistance and a reduced duration of drug use are imperative [19].

Traditional methods for assessing TB treatment efficacy are subjective and time-consuming. Although new biomarkers show promise, their evaluation is still in its early stages. Artificial intelligence (AI) offers a faster, more convenient technique to predict TB treatment efficacy, especially in pulmonary TB (PTB). Through integrating images or clinical data, AI can screen, diagnose, and predict outcomes, enhancing accuracy in medical assessments [20,21].

AI systems are capable of analyzing CT scans and chest X-rays to find TB-related anomalies, like cavities and nodules, that may be signs of MDR or XDR TB. To find drug-resistant TB strains, they can also examine the outcomes of laboratory tests such as drug susceptibility testing (DST), culture, and sputum smear microscopy. Compared to manual approaches, AI helps comprehend complicated patterns and identify medication resistance more quickly and correctly [22,23]. It facilitates the diagnosis of MDR and XDR TB by offering clinicians decision-support tools that take into account patient data, such as symptoms, medical history, and risk factors. Based on the most current guidelines and data, these tools recommend suitable diagnostic procedures and methods for treatment [24]. In the present review, we highlighted the advancements in AI for the diagnosis of MDR and XDR tuberculosis.

## Review

Methodology

The review technique was developed under the guidance of the Preferred Reporting Items for Systematic Review and Meta-Analysis Protocols (PRISMA-P) declaration. All modifications made to the protocol were duly recorded and documented.

Search Strategy

A thorough literature search was carried out across various databases, namely Google Scholar, PubMed, the Cochrane Library, Scopus, Web of Science, Embase, and Wiley. The process of selecting studies adhered to pre-established inclusion criteria, which were attained by carrying out a search employing usual MeSH terms: '#Pulmonary tuberculosis #Artificial intelligence #Multidrug resistance #Extensively drug-resistant tuberculosis #Advantages #Uses/benefits of AI #Machine learning in diagnosis #Early diagnostic approaches #Global burden of tuberculosis #Epidemiology of tuberculosis #Morbidity and mortality rate due to tuberculosis #Drug susceptibility testing (DST), culture, and sputum smear microscopy #Advancement in AI for diagnosis of MDR tuberculosis.'

Inclusion Criteria

Studies published in peer-reviewed journals. Studies focus on the use of artificial intelligence (AI) for the diagnosis of multidrug-resistant (MDR) and extensively drug-resistant (XDR) tuberculosis. Studies that include human subjects. Studies that report on the development or validation of AI models for the diagnosis of MDR and XDR tuberculosis. Studies that compare AI-based methods with conventional diagnostic methods for MDR and XDR tuberculosis. Studies that report on the performance metrics (sensitivity, specificity, accuracy, etc.) of AI models for diagnosing MDR and XDR tuberculosis were included.

Exclusion Criteria

Studies that were not published in English. Studies that do not focus on the diagnosis of MDR and XDR tuberculosis. Studies that focused solely on drug-susceptible tuberculosis. Studies that do not involve the use of artificial intelligence. Studies that are not relevant to the objectives of the review. Studies, including case reports, letters to the editor, short communications, scoping reviews, narrative reviews, and animal studies, were excluded from the present review article.

Study Selection

Using inclusion/exclusion criteria, evaluate the relevancy of titles and abstracts. Retrieve the full texts of potentially relevant studies for detailed evaluation. Employ a systematic approach involving at least two independent reviewers for study selection.

Statistical Analysis

We first conducted a database search and then grouped the chosen articles into an Excel spreadsheet. Then, we removed any articles that were duplicates. Next, the full-text publications and abstracts were examined. Furthermore, we meticulously reviewed each of the publications that were chosen for this study before deciding which ones to use. Additionally, we evaluated the included studies using RevMan 5.4 to analyze the risk of bias, study design, and methodological quality.

Results

**Figure 1:**
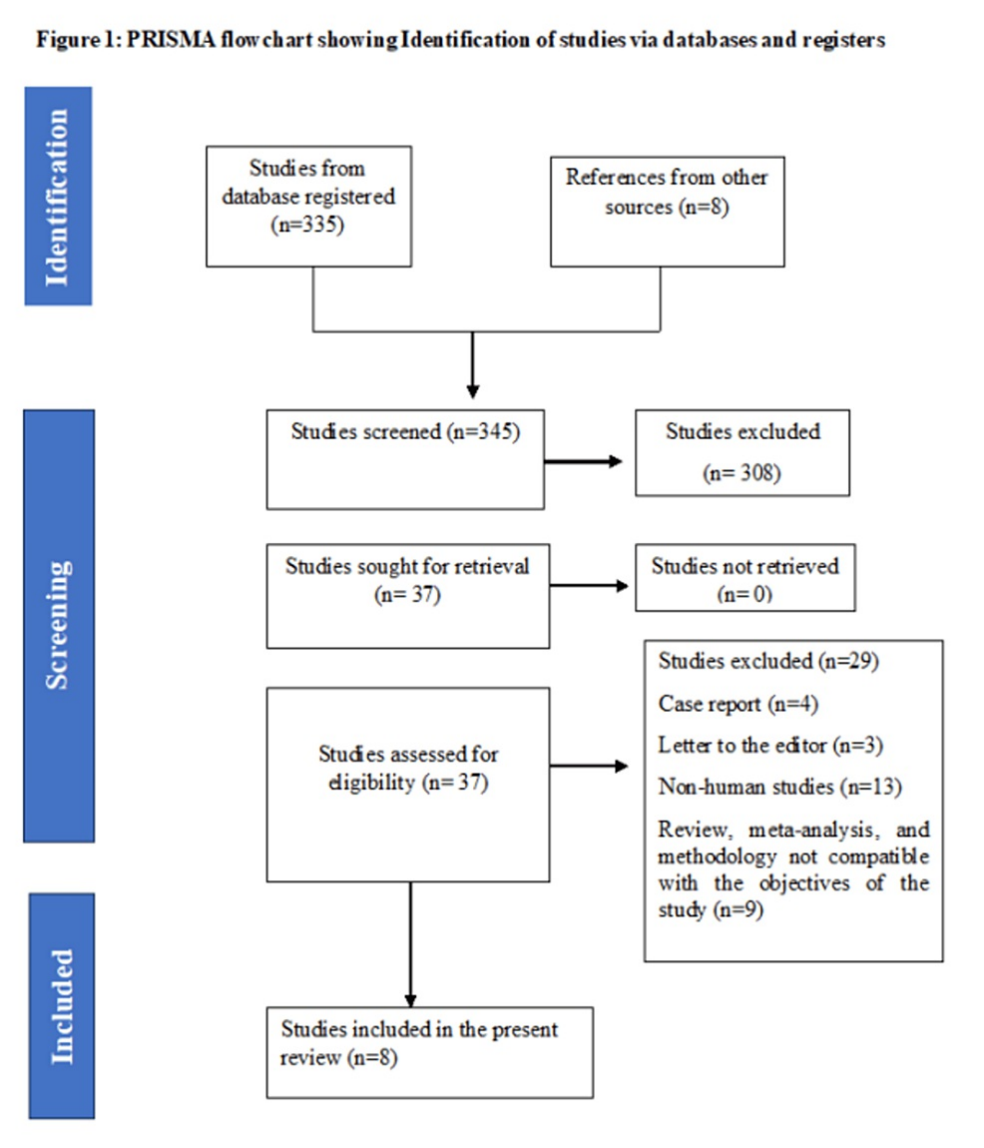
PRISMA-P flow chart PRISMA-P: Preferred Reporting Items for Systematic Review and Meta-Analysis Protocols

In this comprehensive systematic review, an initial screening of 335 studies was conducted, followed by the inclusion of eight additional studies from supplementary references. Then, approximately 345 articles underwent screening based on their titles and abstracts, resulting in the exclusion of 308 studies. A total of 37 full-text articles or studies were then meticulously assessed for eligibility, leading to the exclusion of 29 articles. Exclusion criteria were applied based on article type, including studies lacking information on autoimmune diseases, case reports or studies with small sample sizes, letters to the editor, reviews, meta-analyses, and studies not published in English. After these, through examination or screening, approximately eight studies were finally selected for inclusion in the systematic review, following the predefined inclusion and exclusion criteria (Figure 1).

Similarly, Table 1 provides detailed information about the patient characteristics of the included studies.

**Table 1:** Detailed information about the patient characteristics of the included studies TB: tuberculosis; AI: artificial intelligence; rpoB: RNA polymerase β subunit; inhA: Enoyl acyl carrier protein reductase; KatG: Catalase-peroxidase enzyme; F: Random Forest; CNN: Convolutional neural networks; CT: Computed tomography; SVM: support vector machine; AUC: the area under the ROC; ROC: Receiver operating characteristic curves; DCNN: Deep convolutional neural network; CXR: Chest X-ray.

Author	Country	Mean age	Cases	Algorithm	Sensitivity (TB AI)	Specificity (AI)	Primary material	Final outcome
Xiong Y, et al. (2018) [25]	China	-	201	CNN model, named TB AI (TB-AI),	97.94%	83.65%	Sociodemographic characteristics and clinical parameters	TB-AI aids in TB bacilli detection and clinical decisions, reducing pathologists' workload and missed diagnoses.
Yan C, et al. (2022) [26]	China	48.5 ± 16.5	526	AI-based fully automated CT image	-	-	Demographic profile and lung lesions	The AI system based on chest CT can match human diagnostic performance, aiding early TB detection and optimal patient care.
Balakrishnan V, et al. (2023) [27]	Malaysia	-	-	SVM, Linear Regression, Lasso Regression, Ridge Regression, RF, and Gradient Boosting Regression	-	-	Sociodemographic characteristics and clinical parameters	The findings of this study shows, the efficacy of machine learning in predicting TB treatment duration using risk factors."
Jamal S, et al. (2020) [28]	India			Naïve bayes, k nearest neighbor, support vector machine, and artificial neural network			A total of 130, 237, 11, 263, 17, and 16 variations were obtained for rpoB, pncA, inhA, katG, gyrA, and gyrB, respectively.	
Higashiguchi M, et al. (2021) [29]	Japan	67 years	239	CNNs	-	-	Posteroanterior X-ray, radiographs in the standing position	The CNN model effectively predicted culture negativity duration in active pulmonary TB, but with unsatisfactory accuracy. The study highlights the importance of chest radiography findings alongside clinical factors for machine learning.
Nijiati M, et al. (2022) [30]	China		9,628 CXR images	CNNs	93.2-95.5%	95.78-98.05%		The ResNet algorithm-based AI diagnosis system accurately diagnosed TB, showing potential for clinical use, especially in high TB incidence, low-resource regions.
Rajaraman S, et al. (2022) [31]	South Africa		677	Deep learning	0.67%	0.87%		
Lakhani P, et al. (2017) [32]	United States, China, and Belarus		1,007	CNNs	97.30%	94.70%		Using deep learning with DCNNs achieves accurate TB classification in chest radiography, with an AUC of 0.99. Incorporating radiologist input for cases of classifier disagreement further enhances accuracy.

**Figure 2:**
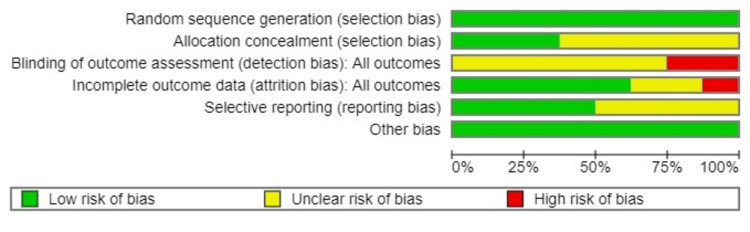
A summary of the risk of bias for the prevalence of studies conducted.

**Figure 3:**
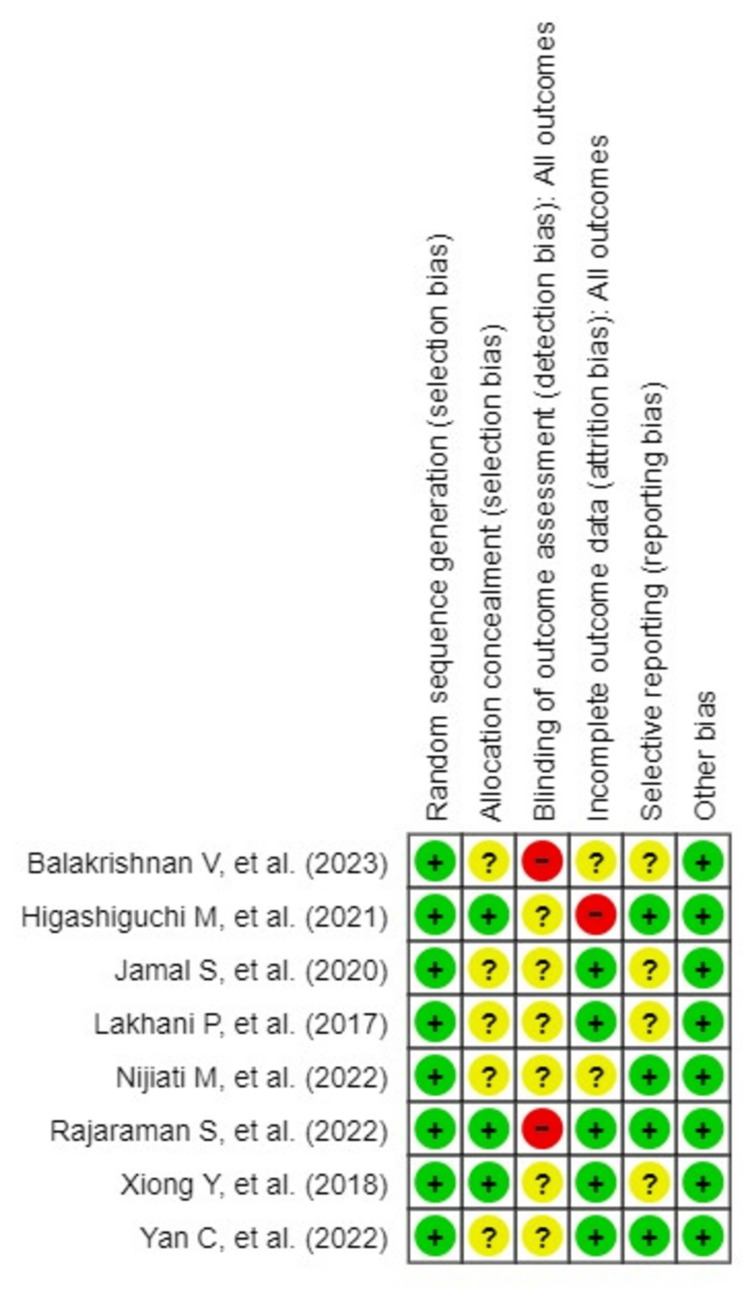
A summary of the risk of bias for the prevalence of studies conducted.

A summary of the risk of bias for the prevalence of conducted studies is shown in Figures 2, 3.

In the present review, approximately 12,278 cases were studied, and CNNs were the most common algorithm used. According to the various study outcomes, TB-AI is proven to be an effective tool for the detection of *TB bacilli*.

Discussion

This review examines AI's role in predicting TB treatment effectiveness over the past decade. While computer technology aids in TB diagnosis, AI applications primarily focus on this aspect. Early TB diagnosis is crucial due to MTB's extended incubation period. Additionally, predicting and adjusting ATTB treatment efficacy is vital for improving cure rates and preventing transmission [20,33,34].

AI models like Random Forest (RF), convolutional neural networks (CNN), support vector machines (SVM), and linear regression (LR) were researched for predicting treatment duration, efficacy, and DR of ATTB therapy. Current studies highlight CNN's low mean absolute error (MAE), making it a preferred choice for guiding clinicians in determining individual treatment durations for PTB patients [29]. Genes as variables show significant potential in predicting PTB treatment duration, with an area under the receiver operating characteristic curve (ROC) value of up to 0.94 [35].

According to some investigations, predicting TB treatment duration using AI and machine learning is limited, necessitating improvements in the robustness and popularity of existing models. Models like RF, XGBoost, LR, ANN, RP, Bagging, and SDLM show over 80% accuracy in predicting treatment outcomes and adverse reactions. SVM, though slightly less effective, still achieves over 70% accuracy, indicating significant clinical potential. Model accuracy varies based on the included parameters and variables. For instance, the RF model, according to Liao KM et al., reaches 85.6% accuracy [36], while Rosenfeld G et al. report a range of 74% to 84% accuracy [37]. Despite high specificity, addressing low sensitivity (<50%) is crucial. MTB resistance prediction is a key focus, with DCNN and CNN+SVM models surpassing 80% accuracy. SVM, RF, multi-label K-nearest neighbor (MLKNN), ensemble of classifier chain (ECC), and deep denoising auto-encoder (DeepAMR) models achieve an AUC value exceeding 0.9, showing promising applications. Li Y et al.'s nodule model and combined model (TIB and nodules), based on the RF classifier, exhibit exceptional performance with an accuracy (>80%) and AUC (>0.9) [38].

Current research trends involve adjusting model parameters and variables and employing multiple models to enhance prediction accuracy. AI in ATTB treatment currently prioritizes adverse reactions and outcomes but lacks focus on predicting treatment duration. While forecasting adverse reactions and results is crucial, the standard six-month isoniazid regimen may not suit every case, leading to potential issues. Prolonged treatment can impact compliance and efficacy, resulting in increased risks [39]. Recent interest in using genomic techniques and CT imaging for predictive models has grown, leveraging diverse data types. Enhancements in predictive modeling incorporating more biomarkers and clinical data can improve accuracy. Validating with independent sample sets ensures model universality [40]. Integrating AI into clinical decision-making requires effective guidelines for translating results into actionable recommendations, considering individual patient differences. Future studies should aim for continuous improvement in high-precision models, comprehensive validation, and actionable guidelines for practical implementation in clinical practice [38]. Further, to achieve TB elimination, efforts to bring newer techniques for diagnosis and management are essential. In high-burden centers, these newer modalities could bring remarkable improvements in the management algorithms.

AI in PTB treatment monitoring has limitations, primarily ethical concerns like privacy breaches. While AI predicts efficacy, issues of informed consent and data storage arise. The imperfect accuracy of algorithms raises accountability questions for potential harm [20]. Skepticism among clinicians and patients results from a limited understanding of AI in this field. Addressing ethical concerns requires prioritizing patient data privacy, confidentiality, fairness, and transparency. Establishing a secure medical data system and governance framework is crucial. Validation in diverse populations and continual refinement are needed for robust, adaptable models.

## Conclusions

In summary, this review investigates the progress in AI, particularly within machine learning and deep learning, demonstrating a substantial enhancement in the precision of drug-resistant tuberculosis diagnosis. These advancements exhibit considerable potential for swift and dependable detection, allowing for more individualized treatment strategies and a reduction in the dissemination of drug-resistant strains. Ongoing collaboration between the medical and AI sectors is imperative to refine models and address practical challenges. Eventually, AI represents a promising and efficacious strategy for confronting the worldwide challenge posed by drug-resistant tuberculosis.
